# The Treatment of Obstinate Ringworm

**Published:** 1901-11-16

**Authors:** 


### THE TREATMENT OF OBSTINATE RINGWORM.

Dr. Phineas Abraham [1] says that in the treatment of ringworm one has to try to destroy the fungus and to render the soil unsuitable for its growth, all of which is quite easy if, as in tinea circinata of the body, the fungus is superficial or not too far below the surface of the skin. Anything will do—tincture of iodine, turpentine, sulphurous acid, or even common ink. But tinea of the scalp is not always so easily cured unless it is attacked at the very beginning, it being very difficult to get at the fungus when it has got deeply into the hair follicles. In obstinate cases what Dr. Abraham does is first to epilate as many of the hairs as possible, then to have the scalp washed first with hot water and soft soap, and afterwards with alcohol to remove as much fatty matter as possible, and then he applies an apparatus of the nature of an air pump, by which he exhausts the air from the follicles of the scalp and then allows a penetrating germicide to flow in under atmospheric pressure. The germicide which he uses is a mixture of creasote with tincture and liniment of iodine. In addition to doing this he instructs the friends to rub in with a stiff brush night and morning, an ointment containing a drachm of carbolic acid and a drachm of salicylic acid to the ounce of vaseline. One of the ways in which ointments do good in such cases is, he says, by preventing the access of air, for the fungi require air for their growth ; another is that the ointments prevent the fungus from getting distributed, so that it is well that some of the ointment should be pretty widely spread over the scalp. Twice a week the scalp is to be washed with soft soap containing carbolic and boric acid (a drachm of each to two ounces of soft soap). Then the above-mentioned machine can be used for the patches, and then the ointment thoroughly applied all over. Treatment must be continued till every trace of disease has gone, *i.e.* so long as there is the least degree of scurfiness left, or a single stumpy hair to be found. The use of the microscope is all important in diagnosis, especially where a generalised scurfy condition of the scalp has developed from the running together of the patches, the scalp sometimes becoming so thickly covered with scurf that the short hairs may be scarcely if at all visible. By scraping off some of the scurf and placing it under the microscope with a little liquor potassæ the fungus is soon seen in abundance.

[1] Clin. Jour., Oct. 23.

